# Oocyte TET3: an epigenetic modifier responsible for maternal inheritance of glucose intolerance

**DOI:** 10.1038/s41392-022-01170-0

**Published:** 2022-10-08

**Authors:** Xiaoxue Jiang, Fei Xiao, Feifan Guo

**Affiliations:** Jinshan Hospital, State Key Laboratory of Medical Neurobiology, Institute for Translational Brain Research, MOE Frontiers Center for Brain Science, Fudan University, Shanghai, China

**Keywords:** Metabolic disorders, Epigenetics

In a recent paper published in *Nature*, Chen et al. provide a new perspective on how glucose tolerance of the offspring is affected by pregestational hyperglycaemia.^1^ They showed that oocytes isolated from hyperglycemic mice transmit the impaired glucose homeostasis to the offspring via epigenetic modifications, and insufficiency of oocyte TET3 dioxygenase is indispensable for this process.

The prevalence of diabetes has increased substantially worldwide. It not only damages people’s health, but also burdens economic development. It has become an important social problem that needs to be solved urgently. Studies have shown that maternal hyperglycaemia increases the risk of long-term metabolic diseases in the offspring. Most studies investigate the association between gestational diabetes and metabolic syndrome in the offspring, especially the effects of the maternal environment during pregnancy; however, little information exists on the link between pregestational diabetes and offspring health. It is interesting and crucial to investigate whether and how the alterations in maternal germ cells affect metabolic phenotypes in the offspring. Several studies have demonstrated that sperm influence offspring via epigenetic mechanisms, such as DNA methylation, histone modification and small RNAs.^2^ These reports indicate that maternal germ cells may have similar functions. Although there is limited literature, observations from a previous study support this hypothesis.^3^ To distinguish the effects of the in utero environment from altered factors in the oocyte, Huypens et al. isolated oocytes from high-fat diet (HFD) mice and used in vitro fertilization (IVF) technology. They found that offspring derived from HFD-exposed oocytes were more likely to become obese and glucose intolerant than those from mice fed a normal diet. However, the authors did not identify the factors that had changed in the oocytes or the underlying mechanisms. Recently, a study published in *Nature* has investigate these issues.^1^

**Fig. 1:**
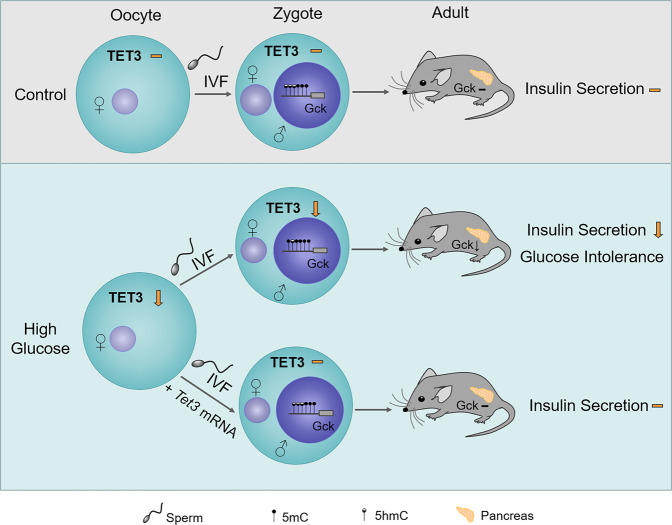
Oocyte TET3 mediates maternal inheritance of glucose intolerance. The mature sperm genome undergoes active demethylation after fertilization. Oocyte TET3 is responsible for demethylation of the paternal genome by catalyzing the conversion of 5mC into 5hmC and other high-oxidation products. In fertilized oocytes from healthy mice, TET3 demethylates the promoter of several genes involved in insulin secretion such as *Gck*, leading to normal expression of these genes and insulin secretion in the pancreas at the adult stage. In contrast, *Tet3* mRNA is reduced in oocytes from hyperglycemic mice, resulting in hypermethylation of the paternal-derived *Gck* promoter. This leads to decreased *Gck* expression and lower levels of insulin secretion in the pancreatic islets of the offspring in adulthood. Furthermore, injection of exogenous *Tet3* mRNA in oocytes from hyperglycemic mice could rescue impaired insulin secretion, and thus glucose intolerance in offspring

To uncover whether oocytes could transmit pregestational hyperglycaemia to the next generation, Chen et al. isolated oocytes from streptozotocin (STZ)-induced hyperglycaemia female mice, fertilized these oocytes with sperm from healthy male mice in vitro, and transferred them into healthy foster mothers to produce offspring (Fig. 1). This approach is beneficial as it excludes variables related to gestation and lactation, ensuring that any inherited phenotype is exclusively transmitted through oocytes. They observed that the offspring derived from the hyperglycemic females exhibited impaired glucose tolerance, probably due to defective insulin secretion by pancreatic beta cells. These results suggest that preconception health management can protect offspring health. Interestingly, Chen et al. found that the effects of maternal pregestational hyperglycaemia are unlikely to persist to the second generation (at least 24-week-old).

To evaluate the underlying mechanisms, Chen et al. performed RNA sequencing on oocytes from hyperglycemic mice and controls, and TET3 was notable for its high abundance and fold change. TET3 is a dioxygenase that belongs to the ten-eleven translocation (TET) gene family. It catalyzes the conversion of 5-methylcytosine (5mC) into 5-hydroxymethylcytosine (5hmC), and other high-oxidation products, thereby playing a role in DNA demethylation.^4^ *Tet3* mRNA was found to be reduced in oocytes from hyperglycemic mice and humans with diabetes, leading to impaired zygotic DNA demethylation in certain regions such as promoters. Particularly, TET3 mainly facilitated paternal genome demethylation. Subsequently, the authors conducted methylome analysis of mouse embryonic pancreatic islets to search for downstream targets of TET3. As promoter methylation is usually inversely associated with transcription, the authors focused on hypermethylation at the promoter. They found that several genes with hypermethylation at the promoter were enriched in the insulin secretion pathway, including the glucokinase gene (*Gck*). Glucokinase plays a key role in insulin secretion. The authors observed increased methylation of the paternal-derived *Gck* promoter throughout mouse development, from zygote to adult. This resulted in decreased *Gck* expression and lower levels of insulin secretion in the pancreatic islets of offspring from hyperglycemic mice at the adult stage. Similar hypermethylation at the *Gck* promoter was observed in blastocysts from human couples in which the woman had diabetes. Of note, consistent with the promoter hypermethylation, mRNA expression of several other genes—besides *Gck*—involved in the insulin secretion pathway were also reduced in adult pancreatic islets owing to oocyte TET3 insufficiency.

To further confirm the role of TET3 in oocyte, Chen et al. generated oocyte-specific *Tet3* knockout female mice. They observed increased methylation levels of the *Gck* promoter in zygotes from these mice and in pancreatic islets from their offspring. Consistently, the offspring of these mice exhibited impaired glucose tolerance. Moreover, injection of exogenous *Tet3* mRNA in oocytes from hyperglycemic mice was sufficient to rescue the maternal effect on glucose intolerance in the offspring. These data indicate that oocyte TET3 mediates pregestational hyperglycaemia-impaired glucose tolerance in offspring. This research has huge translational application value. For early diagnosis, TET3 can be used as a biomarker to predict the development of diabetes in the offspring. For preconception interventions, nutritional or pharmacological modulation of TET3 expression may be a feasible approach to alleviate metabolic disorder in the offspring.

Taken together, this study identifies TET3 as a key epigenetic factor in the oocyte responsible for transmitting glucose intolerance from mother to offspring. These findings provide revolutionary new insights for the prevention of diabetes at the oocytes stage, which may help to reduce the occurrence of metabolic diseases in the next generation. This study also expands the understanding of the role of TET3 in metabolic functions. There are limited reports about the effects of TET3 on metabolism. It has been shown that TET3 promotes hepatic glucose production, and hepatic knockdown of TET3 improves glucose homeostasis in dietary and genetic mouse models of diabetes.^5^ This study and the work of Chen et al. suggest that TET3 in different organs exerts distinct effects on whole-body glucose metabolism.

We would like to discuss some potential limitations of this study and future perspectives. First, although insufficient insulin secretion in pancreatic islets contributes to glucose intolerance here, alterations in other tissues may also affect offspring glucose metabolism. Therefore, it is worth investigating changes in other tissues. Second, although the modulation of DNA methylation is important for maternal inheritance, other mediators such as RNAs and mitochondria in oocytes may also have lasting effects on the offspring. It would be interesting to explore the impact of other alternative factors. Third, TET3 is widely expressed and have many other physiological functions, such as neurogenesis. It is crucial to evaluate whether its insufficiency in oocytes may affect the neurogenesis of offspring.
